# Selection and characterization of a promoter for expression of single-copy recombinant genes in Gram-positive bacteria

**DOI:** 10.1186/1472-6750-5-3

**Published:** 2005-01-14

**Authors:** Roberta Provvedi, Tiziana Maggi, Marco R Oggioni, Riccardo Manganelli, Gianni Pozzi

**Affiliations:** 1 Laboratory of Molecular Microbiology and Biotechnology, Department of Molecular Biology, University of Siena, Policlinico "Le Scotte", Viale Bracci, 53100 Siena, Italy; 2 Department of Histology, Microbiology and Medical Biotechnologies, University of Padova, Medical School, Via A. Gabelli 63, 35121 Padova, Italy; 3 IRIS Research Center, Chiron S.r.l., Via Fiorentina 1, 53100 Siena, Italy

## Abstract

**Background:**

In the past ten years there has been a growing interest in engineering Gram-positive bacteria for biotechnological applications, including vaccine delivery and production of recombinant proteins. Usually, bacteria are manipulated using plasmid expression vectors. The major limitation of this approach is due to the fact that recombinant plasmids are often lost from the bacterial culture upon removal of antibiotic selection. We have developed a genetic system based on suicide vectors on conjugative transposons allowing stable integration of recombinant DNA into the chromosome of transformable and non-transformable Gram-positive bacteria.

**Results:**

The aim of this work was to select a strong chromosomal promoter from *Streptococcus gordonii *to improve this genetic system making it suitable for expression of single-copy recombinant genes. To achieve this task, a promoterless gene encoding a chloramphenicol acetyltransferase (*cat*), was randomly integrated into the *S. gordonii *chromosome and transformants were selected for chloramphenicol resistance. Three out of eighteen chloramphenicol resistant transformants selected exhibited 100% stability of the phenotype and only one of them, GP215, carried the *cat *gene integrated as a single copy. A DNA fragment of 600 base pairs exhibiting promoter activity was isolated from GP215 and sequenced. The 5' end of its corresponding mRNA was determined by primer extention analysis and the putative -10 and a -35 regions were identified. To study the possibility of using this promoter (PP) for single copy heterologous gene expression, we created transcriptional fusions of PP with genes encoding surface recombinant proteins in a vector capable of integrating into the conjugative transposon Tn*916*. Surface recombinant proteins whose expression was controlled by the PP promoter were detected in Tn916-containing strains of *S. gordonii *and *Bacillus subtilis *after single copy chromosomal integration of the recombinant insertion vectors into the resident Tn*916*. The surface recombinant protein synthesized under the control of PP was also detected in *Enterococcus faecalis *after conjugal transfer of a recombinant Tn*916 *containing the transcriptional fusion.

**Conclusion:**

We isolated and characterized a *S. gordonii *chromosomal promoter. We demonstrated that this promoter can be used to direct expression of heterologous genes in different Gram-positive bacteria, when integrated in a single copy into the chromosome.

## Background

In the past ten years there has been a growing interest in engineering Gram-positive bacteria for biotechnological applications, including vaccine delivery. [1-4], and *in situ *production of anti-infective protectants [5] and microbicides [6]. A common approach to genetic manipulation of bacteria is based on the use of plasmid expression vectors since these recombinant molecules can be introduced into bacterial cells by a variety of genetic techniques such as natural transformation, artificial transformation, transduction, conjugative mobilization, and electroporation [7-9]. However, the major limitation of this approach is due to the fact that recombinant plasmids are often lost from the bacterial culture upon removal of antibiotic selection. Certainly, this has consequences when using recombinant bacteria *in vivo *where their replication occurs in the absence of selection. An alternative approach is to integrate recombinant DNA molecules into the bacterial chromosome since this method allows increased *in vivo *stability of the genetic constructs. Therefore a lot of efforts have focused on the development of efficient expression systems based on chromosomal integration of expression cassettes [10,11]. Naturally transformable bacteria represent a convenient model, since heterologous DNA can be easily integrated into their chromosomes, whereas genetic manipulation of non-transformable bacteria is more difficult and relies mainly on electroporation and conjugative mobilization of foreign DNA molecules.

We have previously described a genetic system based on conjugative transposons allowing stable integration of recombinant DNA into the chromosome of transformable and non-transformable streptococci [12,13]. A series of transposon insertion vectors containing two regions of homology with Tn*916 *[14] have been created in order to manipulate both naturally transformable and non-transformable Gram-positive bacteria carrying Tn*916 *[12]. The aim of this work was to select a strong promoter to improve this genetic system making it suitable for expression of single-copy recombinant genes in a broad spectrum of Gram-positive bacteria.

## Results and discussion

### Promoter selection by chromosomal integration

**Figure 1:**
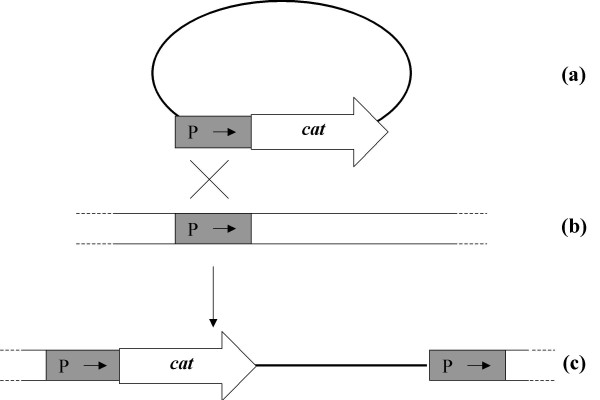
***In vivo *transcriptional fusion by integration of a promoterless *cat *gene into the chromosome of a naturally transformable streptococcus**. A promoterless *cat *gene is ligated *in vitro *to random chromosomal fragments, using a restriction site a few base pairs upstream of its translation initiation codon (a). The ligation mixture is then used to transform the recipient strain (b), and the homologous sequences allow chromosomal integration of the promoterless *cat *gene (c). By this process, the *cat *gene is integrated into the chromosome between two direct repeats. Since some of the chromosomal fragments contain promoters (P), it is possible to obtain expression of the promoterless *cat *gene after *in vivo *transcriptional fusion with resident chromosomal promoters.

To select resident promoters from the genome of *Streptococcus gordonii*, we performed a random ligation of streptococcal DNA to a promoterless *cat *gene, conferring resistance to chloramphenicol (Cm). The ligation mixture was used to transform the naturally transformable *S. gordonii *«Challis» strain V288 and transformants were selected for Cm resistance. Chromosomal DNA flanking the promoterless *cat *gene provided the homology for the random integration of *cat *into the chromosome during transformation (Fig. 1). 71 Cm-resistant (Cm^R^) transformants were isolated, presumably as a result of transcriptional fusions of streptococcal promoters to the promoterless *cat *gene. Eighteen Cm^R ^transformants were selected for further characterization. The strategy commonly used to select promoters is based on cloning random chromosomal DNA fragments in a promoter probe vector upstream of a promoterless reporter gene. However, integrating the promoterless reporter gene (*cat*) directly into the streptococcal chromosome, allowed us to select resident chromosomal promoters expressing *cat *after *in vivo *transcriptional fusion at a single locus on the chromosome. This is preferable when looking for promoters to express heterologous genes integrated into the chromosome in a single copy.

*In vivo *analysis of promoter strength was determined in the eighteen selected transformants by measuring the minimum inhibitory concentration (MIC) of Cm. Fifteen transformants exhibited a MIC of 16 μg/ml, whereas the remaining three transformants exhibited a MIC of 8 μg/ml. The fifteen strains with higher MIC were tested for the stability of the chloramphenicol-resistance phenotype: after 50 generations of growth without selection, bacterial cultures were plated on non-selective medium and at least 200 colonies were picked and tested for Cm resistance. The stability of the resistance phenotype varied considerably among the different transformants (data not shown). Three strains (GP214, GP215 and GP216) showed a 100% stability and were chosen for further analysis.

### Cloning of a promoter from *S. gordonii*

The structure of the integrated *cat *gene in GP214, GP215 and GP216 was analyzed by Southern blot. Only GP215 showed to have a single *cat *copy integrated into the chromosome, whereas in GP214 and GP216 integration occurred at multiple sites (data not shown). In order to clone the regions flanking *cat *integration site in GP215, the chromosome of this strain was cut with *Taq*I whose recognition sequence is absent inside the *cat *gene. The derivative fragments were ligated to pBLUESCRIPT, and the ligation mixture was used to transform *Escherichia coli *cells; transformants were then selected for Cm^R^. All transformants analyzed for plasmid content showed to carry a plasmid of the same size. One of these transformants (GP334) was selected for further analysis and the transforming plasmid was named pVMB5. By restriction analysis we showed that pVMB5 contains a 2.2 kb *Taq*I insert where a 600 base pairs (bp) streptococcal DNA fragment was cloned upstream of the *cat *gene. This DNA fragment was stably maintained in *E. coli*, where it retained its promoter activity, conferring Cm^R ^to *E. coli*. This is of considerable interest since it is known that very often cloning streptococcal promoters in a high copy number plasmid results in the failure of that plasmid to be established [15].

### Sequence analysis

**Figure 2:**
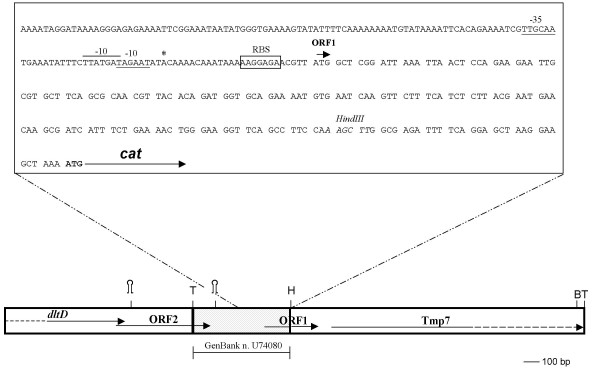
**Schematic representation of the *S. gordonii *locus containing PP promoter**. DltD orthologue, an ORF encoding a putative acetyltransferase (ORF2), ORF1, the gene encoding a Tmp7 transmembrane protein, and the *cat *gene are indicated by arrows. Dashed arrows designate that the ORF is only partially represented in the scheme. The 600 bp *Taq*I-*Hind*III fragment including PP promoter and cloned upstream of the *cat *gene in pVMB5 is indicated by a dotted box. Nucleotide sequence of the PP promoter region (332 bp) is reported inside the box. The transcriptional start site determined by primer extention analysis is marked with an asterisk. Proposed -35 and -10 regions are underlined. A second putative -10 sequence is overlined. ORF1 putative ribosome binding site (RBS) is boxed. ATG initiation codon of *cat *is in bold characters and the sequence of *Hind*III site is in italic letters. The complete sequence of the 600 bp cloned fragment is available on GenBank (Accession number: U74080). T, *Taq*I site; B, *Bam*HI site; H, *Hind*III site ; loop, putative transcriptional terminator.

The streptococcal DNA upstream of the *cat *gene in pVMB5 was sequenced (GenBank accession number: U74080). Analysis of the sequence revealed the presence of an open reading frame (ORF1), preceded by a typical ribosome binding site (RBS) (Fig. 2). A BLAST search with the partial sequenced genome of *S. gordonii * showed that ORF1 represents the 5'-end of a gene encoding the first 48 amino acids (aa) of an uncharacterized protein conserved in bacteria and whose function is unknown. As indicated in figure 2, this truncated streptococcal protein is translationally fused to the N-terminus of CAT. Sequence analysis also revealed that the first 122 bp of the cloned streptococcal fragment belong to the 3'-end of a gene encoding a putative acetyltransferase (ORF2). To obtain information about the chromosomal region containing the 600 bp streptococcal DNA fragment cloned in pVMB5, we looked throughout the whole contig sequence and we found out that upstream ORF2, and partially overlapping with it, there is an ORF encoding a DltD horthologue (*dltD*), a protein involved in D-alanine incorporation into lipoteichoic acid (LTA) [16], whereas downstream ORF1, there is a gene encoding a Tmp7 transmembrane protein (Fig. 2).

### Identification of the transcriptional start site

**Figure 3:**
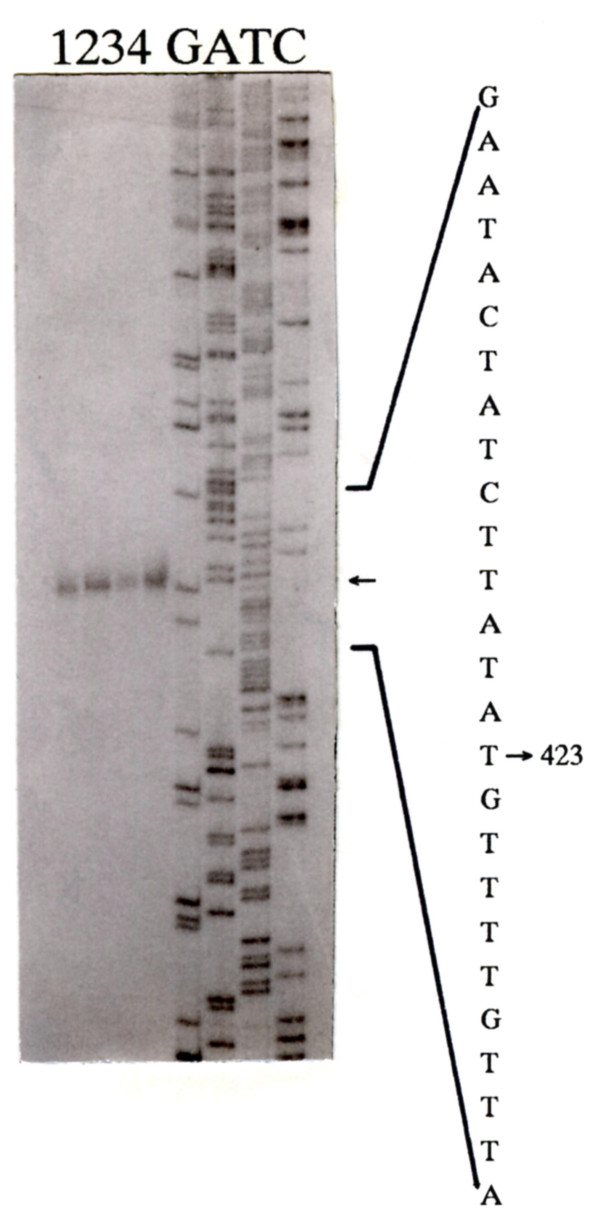
**Primer extension analysis of the PP promoter**. Localization by primer extension of the transcriptional start site of the *cat *mRNA specified by the PP promoter in *E. coli *GP334 (lanes 1–2), and *S. gordonii *GP215 (lanes 3–4). The sequence of the region upstream of the *cat *gene in pVMB5 was used as standard. The A residue, complementary to the T at position 423, indicated by an arrow, represents the transcriptional start site used in both strains.

To identify the sequences responsible for the observed promoter activity, the 5'-end of the *cat-*specific mRNA was mapped by primer extension with a specific primer. Total RNA was isolated from *E. coli *GP334 (containing pVMB5) and *S. gordonii *GP215. In both strains, the position of the 5'-end of the mRNA was located at the same purine residue at position 423 of the 600 bp region of streptococcal DNA, 26 nucleotides upstream of the ORF1 translational start site (Fig. 2 and 3). Putative -35 and -10 sequences closely resembling the consensus *E. coli *σ^70 ^and *Bacillus subtilis *σ^43 ^[17] recognition sequences TTGACA (-35) and TATAAT (-10) could be identified. The -35 region was TTGCAA, and the -10 region was TAGAAT. The spacing between the -35 and -10 region was 17 bp and was thus similar to the spacing in *B. subtilis *(17 to 19 bp) and *E. coli *(16 to 18 bp) promoters. Moreover, a TG nucleotide pair was found 1 bp upstream of the -10 region; such a structure is typical of Gram-positive bacteria promoters [18]. Based on this information, we concluded that indeed we isolated a streptococcal promoter which was designated PP. The spacing between the 5'-end of the mRNA and the -10 hexanucleotide was only 2 bp, which is unusually short. A second putative -10 region could be identified in the TATGAT hexanucleotide (Fig. 2), whose distance from the 5' end of the mRNA is 7 bp. However, since this sequence is not preceded by the TG nucleotide pair typical of Gram-positive promoters, and is separated from the -35 region only by 12 bp, we suppose that this -10 region of PP is probably not active.

### Insertion vectors to express heterologous proteins

**Figure 4:**
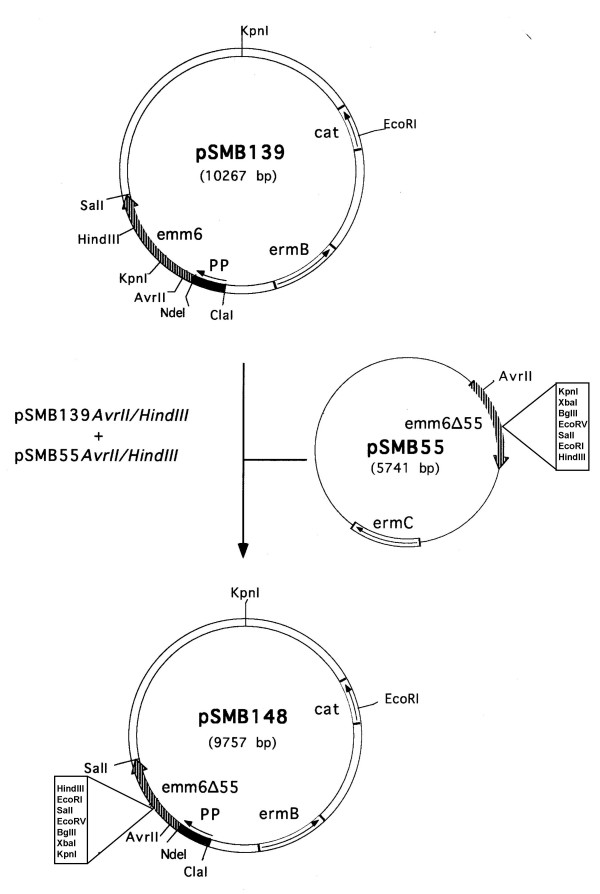
**Construction of the insertion plasmid pSMB148**. pSMB139 was constructed by introducing a 2.0 kb fragment, containing a transcriptional fusion of PP promoter with *emm6*, in the Tn*916 *insertion vector pSMB47 (see Methods). pSMB148 is a derivative of pSMB139 in which a 900 bp *Avr*II-*Hind*III fragment was replaced with a 390 bp *Avr*II-*Hind*III fragment from pSMB55 containing a multiple cloning site [10].

We have previously developed a genetic system based on the use of *Streptococcus pyogenes *surface fibrillar M6 protein, as a partner for the construction of translational fusions to deliver foreign proteins on the surface of *S. gordonii *[10]. To determine the possibility of using PP for chromosomal single copy heterologous gene expression in Gram-positive bacteria, we first generated a transcriptional fusion of PP with a promoterless gene encoding the M6 protein (*emm6*) in pSMB47, a suicide vector capable of integrating heterologous DNA into the conjugative transposon Tn*916 *via homologous recombinantion [12]. The recombinant plasmid was named pSMB139 (see Methods) (Fig. 4). To construct a Tn*916 *insertion vector that could be used to express translational fusions between M6 and any heterologous proteins under the PP promoter control, a 900 bp *Avr*II-*Hind*III fragment internal to *emm6 *in pSMB139 was replaced by a 390 bp *Avr*II-*Hind*III fragment of pSMB55 containing a multiple cloning site [10] (Fig. 4). The resulting vector, named pSMB148 (Fig. 4), allows to create translational fusions of heterologous proteins between the first 122 N-terminal aa and the last 140 aa of the M6 protein, which provides sequences necessary for cell wall anchoring.

**Figure 5:**
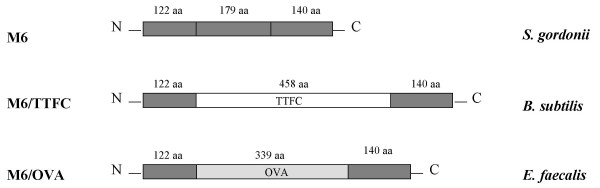
**Schematic representation of recombinant M6, M6/TTFC and M6/OVA expressed on the surface of *S. gordonii*, *B. subtilis *and *E. faecalis***. The 458 aa protein TTFC (white bar) and the 339 aa protein OVA (light gray bar) were fused with the first 122 N-terminal aminoacids and the last 140 C-terminal aminoacids of M6 (dark gray bar). The predicted molecular weight of M6, M6/TTFC and M6/OVA is 49 kDa, 82 kDa and 66.9 kDa respectively.

pSMB148 was used to create two derivative vectors in which the *emm6 *gene was fused respectively with a DNA sequence encoding 339 aa of the chicken ovalbumin (OVA) (pSMB156), and a DNA sequence encoding 458 aa of the tetanus toxin fragment C (TTFC) (pSMB288) (see Methods). A schematic representation of the recombinant proteins is shown in Fig. 5A.

### Expression of recombinant proteins in Gram-positive bacteria

*S. gordonii*, *B. subtilis*, and *Enterococcus faecalis *were three Gram-positive hosts used to analyze the capability of PP to direct transcription of the heterologous genes expressing M6, M6/TTFC and M6/OVA recombinant proteins.

### Expression of M6 in *S. gordonii*

**Figure 6:**
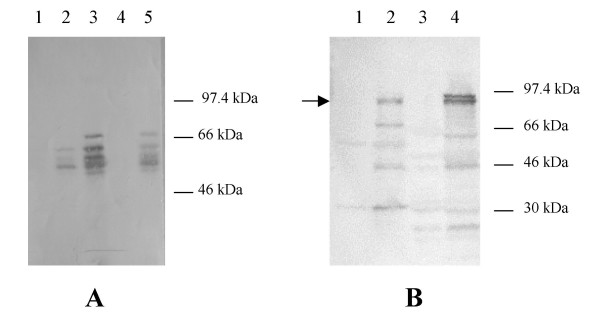
**Western blot analysis of recombinant *S. gordonii *and *B. subtilis *strains expressing M6 protein and M6/TTFC fusion protein**. (A) *S. gordonii *envelope fractions. Lane 1 through 3, GP1241 expressing M6 under the control of PP promoter. Lane 1, GP1241 harvested after overnight growth. Lane 2, GP1241 harvested after early stationary phase. Lane 3, GP1241 harvested after exponential phase. Lane 4, recipient strain GP201 (negative control). Lane 5, GP231 (positive control). Blot was developed with anti-M6 monoclonal antibody 10B6. (B) *S. gordonii *and *B. subtilis *envelope fractions. Lane 1, *S. gordonii *GP204 (negative control). Lane 2, *S. gordonii *GP1253 expressing M6/TTFC (positive control). Lane 3, *B. subtilis *recipient strain GP800.2 (negative control). Lane 4, *B. subtilis *GP848 expressing M6/TTFC under the control of PP promoter. Blot was developed with anti TTFC rabbit serum. Molecular weight markers are shown in the right side of panels.

pSMB139, bearing a transcriptional fusion of PP with *emm6*, was introduced by natural transformation in *S. gordonii *GP201, a strain with a single copy of Tn*916 *integrated into the chromosome. One of the transformants (GP1241), in which the integrative suicide vector drove the integration of the PP-*emm6 *fusion into Tn*916*, was isolated and analyzed for M6 protein expression. Envelope fractions (containing surface associated proteins) prepared from equal amounts of cells grown to mid-log, early and late stationary phase, were analyzed by Western-blotting with an anti-M6 monoclonal antibody. Multiple bands could be detected in fractions of cells grown to mid-log and early stationary phase, whereas no band was detected in the fraction of cultures grown to late stationary phase (Fig. 6A). The intensity of the signal was higher during the mid-log growth phase suggesting that either PP is more active during exponential growth or that the M6 protein is being degraded during stationary phase. The presence of multiple reactive bands of molecular masses close to the hypothetical size of M6 (predicted molecular weight, 49 kDa) is probably due to the fact that coiled-coil proteins like M6 run at aberrant sizes on denaturing gels [19].

### Expression of M6/TTFC in *B. subtilis*

Competent cells of *B. subtilis *GP800.2, containing one copy of Tn*916 *integrated into the chromosome, were transformed with the insertion vector pSMB288, bearing the transcriptional fusion PP-*emm6/ttfc*. One transformant, GP848, was chosen for further studies. A culture of GP848 was grown to mid-exponential phase and analyzed by Western-blotting for the presence of recombinant M6/TTFC. As shown in Fig. 6B, two reactive bands could be detected in the envelope fraction. The lower band, indicated by an arrow, corresponds to the mature protein (predicted molecular weight, 82 kDa), while the upper band probably represents an unprocessed form (predicted molecular weight, 86.4 kDa).

### Expression of M6/OVA in *E. faecalis*

**Figure 7:**
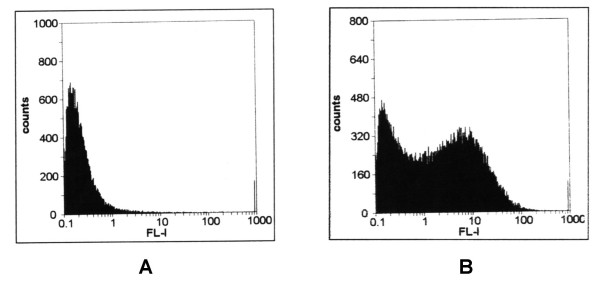
**Flow-cytometric analysis of *E. faecalis *expressing M6/OVA**. (A) OG1SS, recipient strain not expressing M6/OVA. (B) GP431, recombinant strain expressing M6/OVA on the surface. Bacterial cells were treated with anti-OVA rabbit serum and than with FITC-conjugated goat anti-rabbit IgG. *x *axis, arbitrary units (a.u.) of fluorescence intensity (log_10_); *y *axis, relative cell number.

Using a previously described genetic system [12], we constructed a derivative of the *E. faecalis *strain OG1SS [20] expressing the recombinant M6/OVA protein under PP control. pSMB156, bearing the PP-*emm6/ova *fusion, was first introduced in the Tn*916 *containing *B. subtilis *GP800.2 by natural transformation, to obtain a recombinant conjugative transposon containing the transcriptional fusion. The recombinant transposon was then transferred by conjugation into *E. faecalis *OG1SS. Transconjugants were detected at a frequency of 4 × 10^-10 ^transconjugants/recipient. One of them (GP431) was analyzed for cell-surface expression of M6/OVA by flow-cytometric analysis using an anti-ovalbumin polyclonal antibody. The presence of recombinant M6/OVA on the surface of GP431 was clearly demonstrated by the increase of the fluorescence intensity in this strain, as compared to the parental control OG1SS (Fig. 7).

## Conclusions

We have isolated and characterized a promoter from the chromosome of *S. gordonii*, and demonstrated that it can be used to direct expression of heterologous genes in different Gram-positive bacteria when integrated in a single copy into the chromosome. This promoter, together with the genetic system based on suicide vectors able to integrate into conjugative transposons, represents a useful tool for the stable manipulation of a broad spectrum of Gram-positive bacteria.

## Methods

### Bacterial strains, plasmids and growth conditions

All strains and plasmids used in this work are listed in Table 1. *E. coli *strains DH5α and HB101 were cultured in Luria-Bertani (LB) broth. For maintenance of plasmids, ampicillin (100 μg/ml), chloramphenicol (20 μg/ml) or erythromycin (100 μg/ml) was added to the growth medium. Streptococcal strains were cultured in Brain Heart Infusion medium (BHI, Difco) or Tryptic Soy Broth (TSB, Difco) in the presence of chloramphenicol (5 μg/ml), erythromicin (100 μg/ml) or streptomycin (500 μg/ml) whenever required. Transformation of naturally competent cells of *S. gordonii *V288 and GP201, scoring and genetic analysis of transformants was carried out as already described [21,22]. *B. subtilis *strains were grown in LB broth with erythromicin (3 μg/ml) when it was required. Competent cells of *B. subtilis *GP800.2 were prepared and transformed according to described procedures [23]. Agar (1.5%) was added to LB, BHI or TSB to obtain solid media. All cultures were incubated at 37°C.

### DNA manipulation

Total DNA preparation of *S. gordonii *was performed as previously described [21]. Plasmid DNA was prepared using the Qiagen Plasmid Kit (Qiagen) according to the manufacturer's instructions. All recombinant techniques were performed following standard procedures [24], using *E. coli *HB101 or DH5α as a host. DNA restriction enzymes were obtained from Roche and used according to the manufacturer's instructions.

### Promoter selection by chromosomal integration

A 1.6 kb *Hind*III-*Bam*HI fragment of plasmid pKT [25], containing a *cat *gene, was ligated with random chromosomal DNA fragments of *S. gordonii *V288 previously cut with both *Hind*III and *Bam*HI. The initiation codon of *cat *was 34 bp downstream of the *Hind*III site, therefore *Hind*III cleavage would leave *cat *promoterless and preceded by an intact ribosome binding site. The ligation mixture was introduced in *S. gordonii *V288 and transformants were selected for Cm^R^.

### MIC determination

The MIC of chloramphenicol for *S. gordonii *was determined following standard procedures [26].

### Construction of recombinant vectors

A 1648 bp fragment containing the *emm6 *gene (encoding M6, a fibrillar surface protein of *S. pyogenes*) was amplified by PCR from plasmid pVMB20 [22] using the oligonucleotides 5'-ATGGATCCATCATATGGCTAAAAATAACACGAAT-3' (upstream primer, containing a *Bam*HI site and introducing a *Nde*I site at the ATG translation initiation codon) and 5'-GCATGTCGACCATAATCATTAAATGTATCTCAT-3' (downstream primers containing a *Sal*I site). This 1648 bp PCR fragment was digested with *Bam*HI and *Sal*I and cloned in pVA891 [27] previously digested with *Bam*HIand *Sal*I, resulting in plasmid pSMB89. A 451 bp region containing the streptococcal promoter PP was amplified from pVMB5 using the following primers: 5'-CGAGGATCCTTTAATCGATACTCATG-3' (upstream primer, containing a *Bam*HI and a *Cla*I site) and 5'-CCGCATATGGTTCTCCTTTTTATTTGT-3' (downstream primers containing a *Nde*I site). After digestion with *Bam*HI and *Nde*I, this PCR product was inserted between the *Bam*HI and *Nde*I site of pSMB89 to obtain a transcriptional fusion of PP with *emm6*. The resulting plasmid was named pSMB128. This plasmid was first cut with *Bam*HI, treated with Klenow enzyme to generate blunt ends, and finally cut with *Sal*I to obtain a 2.0 kbfragment containing PP-*emm6 *fusion. This fragment was gel-purified and ligated to the suicide integrative plasmid pSMB47 [12] previously cut with *Hind*III, treated with Klenow enzyme to generate blunt ends, and finally cut with *Sal*I. The resulting plasmid was named pSMB139 (Fig. 4). To introduce a multiple cloning site in the *emm6 *gene contained in pSMB139, the 900 bp *Avr*II-*Hind*III fragment internal to *emm6 *was replaced with the 390 bp *Avr*II-*Hind*III fragment of *emm6 *from pSMB55 [10] (Fig. 4). The resulting plasmid was named pSMB148. A 1016 bp DNA region encoding 339 aa of the chicken ovalbumin (OVA) (Gene Bank accession number: V00383) was amplified with the following primers: 5'-CTAGATCTGACAGCA CCAGGACAC-3' (upstream primer containing a *Bgl*II site) and 5'-TAAAGCTTTAGGGG AAACACATCTG-3' (downstream primer containing a *Hind*III site). After digestion with *Bgl*II and *Hind*III, this segment was introduced in pSMB148 previously digested with *Bgl*II and *Hind*III. The resulting plasmid, named pSMB156, contained a translational fusion of M6 with OVA. To create a translational fusion of M6 with the tetanus toxin fragment C (TTFC) a 1374 bp *Bgl*II-*Hind*III fragment encoding 458 aa of TTFC was isolated from pSMB158 [28] and cloned between the *Bgl*II and *Hind*III sites of pSMB148. The resulting plasmid was named pSMB288.

### Western-blot analysis

Preparation of *S. gordonii *and *B. subtilis *cell envelope fractions (representing the protoplast surface containing the cell membrane together with cell wall fragments associated to the protoplasts) was performed as already described [29,30]. The monoclonal antibody 10B6 [31] diluted 1:1000 was used to detect the presence of M6 protein. M6/TTFC fusion protein was visualized with an anti-TTFC rabbit serum (Calbiochem-Novabiochem Corporation) diluted 1:1000.

### Flow-cytometric analysis

Flow-cytometric analysis of *E. faecalis *was performed as already described [28,32] using an anti-OVA rabbit serum diluted 1:300 [29].

### DNA sequence determination

The promoter containing fragment cloned in pVMB5 was sequenced by dideoxy chain termination method [33] as already described [34]. Denatured plasmid DNA was used as template.

### RNA isolation

Total RNA was isolated from a 50 ml cell culture of *S. gordonii *and *E. coli *grown to late exponential phase (OD_590 _≌ 0.5). Cells were harvested by centrifugation at 6000 × *g *at 4°C and lysed according to the following procedures. *E. coli *cells were first resuspended in hot (100°C) lysis buffer (50 mM Tris/HCl pH8, 1 mM EDTA, 1% SDS) and lysed by boiling the suspension for 5 minutes. *S. gordonii *cells were resuspended in lysozyme buffer (25 mM Tris/HCl (pH8), 10 mM EDTA, 50 mM glucose) and subjected to three cycles of freezing in liquid nitrogen and thawing at 52°C. After incubating with 0.2 mg/ml of lysozyme for 30 min at 37°C, one volume of hot (100°C) lysis buffer (100 mM Tris/HCl (pH8), 2 mM EDTA, 2% SDS) was added, and complete lysis was obtained by boiling cells for 5 minutes. After boiling, all lysates were cooled on ice for 5 min and total RNA was purified using the SV Total RNA Isolation System (Promega).

### Primer-extention analysis

Primer extention analysis was performed with a synthetic oligonucleotide 5'-GTTCTTTACGATGCC-3' (position 47 to 61 relative to the initiation of the *cat *gene). Two pmol of the oligonucleotide, labeled with [γ-^32^P] ATP (3000 Ci/mmol, Amersham) using T_4 _polynucleotide kinase (New England Biolabs), were precipitated with 10 μg of RNA and the pellet was resuspended in 8 μl of Moloney Murine Leukemia Virus (M-MuLV) Reverse Transcriptase buffer (50 mM Tris/HCl (pH8.3), 8 mM MgCl_2_, 10 mM DTT). The mixture was heated at 65°C for 3 min, cooled rapidly at -80°C for 1 min and then transfered on ice until it was completely thawed. 1 μl of a 3.75 mM deoxynucleoside triphosphate solution and 10 U of M-MuLV Reverse Transcriptase (New England Biolabs) were added to the RNA-primer hybrid. The reaction mixture was incubated at 48°C for 30 min and terminated with 10 ml of stop solution (95% formamide, 20 mM EDTA pH8.0, 0.05% bromophenol blue, and 0.05% xylene cyanol FF). The reverse transcriptase reactions were analyzed by electrophoresis on a 6% polyacrylamide-7 M urea gel with sequencing reaction obtained with the same primer used as size standards.

### Conjugation

Conjugation experiments were performed on solid media as previously described [12].

## Authors' contributions

RP, characterization of promoter, engineering of *S. gordonii*, writing of manuscript.

TM, engineering of *B. subtilis *and *E. faecalis*

MRO, participation in experimental work, data evaluation

RM, participation in experimental work, data evaluation, writing of manuscript

GP, design and coordination of the study, data evaluation, direct supervision of experimental work, writing of manuscript

All authors read and approved the final manuscript.

## Figures and Tables

**Table 1:** Strains and plasmids used in this work

**Strain**	**Relevant genotype**^a^	**Source/reference**
*S. gordonii*		
V288	Recipient in transformation	[35]
GP201	ΩTn*5253*, Sm^R^, Cm^R^, Tc^R^	[13]
GP204	*str-204*, Sm^R^	[13]
GP214	pKT random in V288, Cm^R^	This work
GP215	pKT random in V288, Cm^R^	This work
GP216	pKT random in V288, Cm^R^	This work
GP231	*emm6*, Er^R^, Sm^R^	[21]
GP1241	ΩTn*5253 *(Δ*tetM*::pSMB139) Er^R^, Sm^R^, Cm^R^, Tc^S^	This work
GP1253	*emm6.1*::TTFC	[28]
		
*E. faecalis*		
OG1SS	Recipient in conjugation, Sm^R^, Sp^R^	[20]
GP431	ΩTn*916*, Er^R^, Tc^S ^(Δ *tetM*::pSMB156)	This work
		
*B. subtilis*		
GP800.2	ΩTn*916*, Tc^R^	[12]
GP847	ΩTn*916*, Er^R^, Tc^S ^(Δ *tetM*::pSMB156)	This work
GP848	ΩTn*916*, Er^R^, Tc^S ^(Δ *tetM*::pSMB288)	This work
		

**Plasmid**	**Description**	**Source/reference**

pKT	Ap^R^, Cm^R^	[25]
pBluescript	Ap^R^	Stratagene
pVMB5	pBluescript::PP-*cat*, Ap^R^, Cm^R^	This work
pVMB20	pBluescript::*emm6.1*::*ermC*, Ap^R^, Er^R^	[21]
pVA891	Cm^R^, Er^R^	[27]
pSMB47	pVA891 derivative containing DNA sequences from Tn*5253*, Cm^R^, Er^R^	[12]
pSMB89	pVA891::*emm6*, Cm^R^, Er^R^	This work
pSMB128	pVA891::PP-*emm6*, Cm^R^, Er^R^	This work
pSMB139	pSMB47::PP-*emm6*, Cm^R^, Er^R^	This work
pSMB148	pSMB47::PP-*emm6/*55, Cm^R^, Er^R^	This work
pSMB156	pSMB47::PP-*emm6/*55::*ova*, Cm^R^, Er^R^	This work

^**a**^Sm, streptomycin; Cm, chloramphenicol; Tc, tetracyclin; Er, erythromycin; spectinomycin; Ap, ampicillin.
